# Naringin vs. *Citrus x paradisi* L. Peel Extract: An *In Vivo* Journey into Oxidative Stress Modulation

**DOI:** 10.3390/antiox14020157

**Published:** 2025-01-28

**Authors:** Jolita Stabrauskiene, Ilona Sadauskiene, Arunas Liekis, Zoja Mikniene, Jurga Bernatoniene

**Affiliations:** 1 Department of Drug Technology and Social Pharmacy, Lithuanian University of Health Sciences, Eiveniu St. 4, LT-50161 Kaunas, Lithuania; jolita.stabrauskiene@lsmu.lt; 2 Neuroscience Institute, Lithuanian University of Health Sciences, Eiveniu St. 4, LT-50161 Kaunas, Lithuania; ilona.sadauskiene@lsmu.lt (I.S.); arunas.liekis@lsmu.lt (A.L.); 3 Large Animal Clinic, Lithuania University of Health Science, Veterinary Academy, LT-44307 Kaunas, Lithuania; zoja.mikniene@lsmu.lt; 4 Institute of Pharmaceutical Technologies, Lithuanian University of Health Sciences, Eiveniu St. 4, LT-50161 Kaunas, Lithuania

**Keywords:** oxidative stress, naringin, naringenin, grapefruit extract, antioxidant biomarkers, GSH, MDA, CAT, aluminum chloride, *Citrus x paradisi* L.

## Abstract

Citrus fruits, mainly grapefruit (*Citrus x paradisi* L.), are rich in bioactive compounds with potential antioxidant properties. This study investigated the antioxidant effects of naringin (NR) and ethanolic *Citrus x paradisi* L. peel (E) in reducing aluminum chloride (AlCl_3_)-induced oxidative stress in mice. Quantitative analysis using HPLC identified optimal extraction conditions, combination ultrasound and reflux extraction (UH50), resulting in high concentrations of naringin (49.13 mg/g) and naringenin (63.99 µg/g). Mice were treated with NR and E to evaluate their effects on key markers of oxidative stress: reduced glutathione (GSH), malondialdehyde (MDA), and catalase (CAT). The E effectively reduced MDA levels in blood, brain, and liver tissues, with a more substantial effect on controlling lipid peroxidation. In contrast, NR was more effective in restoring GSH levels and CAT activity, suggesting a broader enhancement of antioxidant defense. These findings provide information about specific mechanisms of NR and E and their therapeutic potential in managing oxidative stress and developing products with synergistic efficacy.

## 1. Introduction

Oxidative stress is an influential factor contributing to the development of chronic diseases, such as cardiovascular diseases, diabetes, neurodegenerative disorders, and cancer. This condition occurs when the balance between reactive oxygen species (ROS) production and antioxidant defenses is disrupted, damaging essential biomolecules such as lipids, proteins, and nucleic acids [1,2]. Research for effective strategies to reduce oxidative stress has become an essential area of study, focusing on natural antioxidants. For example, curcumin, a bioactive compound derived from turmeric, has shown significant promise in reducing malondialdehyde (MDA) levels and restoring glutathione (GSH) concentrations in oxidative stress models [3]. Similarly, stevia leaf extracts demonstrated the ability to reduce oxidative damage by enhancing enzymatic defenses [4]. These findings provide a comparative basis for evaluating the antioxidant properties of naringin and grapefruit extract, highlighting the need for targeted approaches to modulate oxidative stress.

### 1.1. Naringin and Its Pharmacological Effects

Citrus fruits, mainly grapefruit (*Citrus x paradisi* L.), are well known for their high content of bioactive compounds, such as flavonoids, vitamins, and minerals [5]. Grapefruit extract, derived from the fruit’s pulp and peel, contains a variety of flavonoids, with naringin being one of the most well known.

Regarding the growing interest in both naringin and grapefruit extract, no direct studies have been identified that compare their effects in vivo, particularly regarding their ability to modulate oxidative stress. While several studies have explored their individual antioxidant properties, a side-by-side comparison in controlled in vivo models remains unexplored, highlighting a gap in current research.

**Figure 1:**
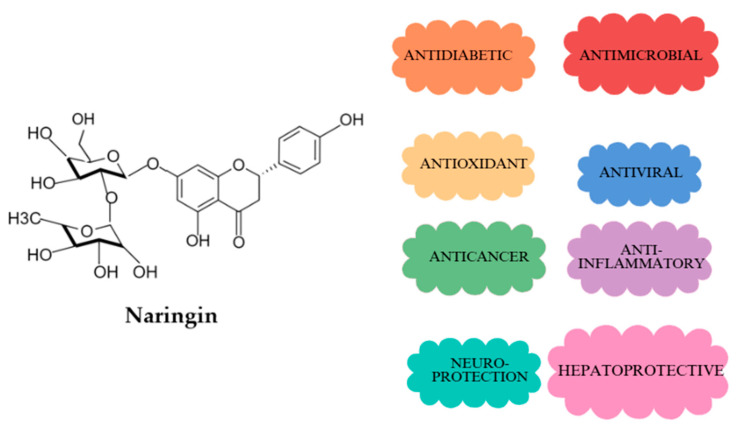
The structure and effects of naringin.

Naringin (4′,5,7-trihydroxyflavanone-7-rhamnoglucoside), as illustrated in Figure 1, belongs to the flavanone class of flavonoids and is mainly found in citrus fruits like lemons, oranges, mandarins, and grapefruits. This essential compound is known for its potent antioxidant, anti-inflammatory, cardioprotective, and anticancer properties [6,7,8,9]. Naringin’s ability to neutralize free radicals and its low toxicity make it a promising candidate for reducing cellular damage caused by oxidative stress [10].

### 1.2. Free Radicals

**Figure 2:**
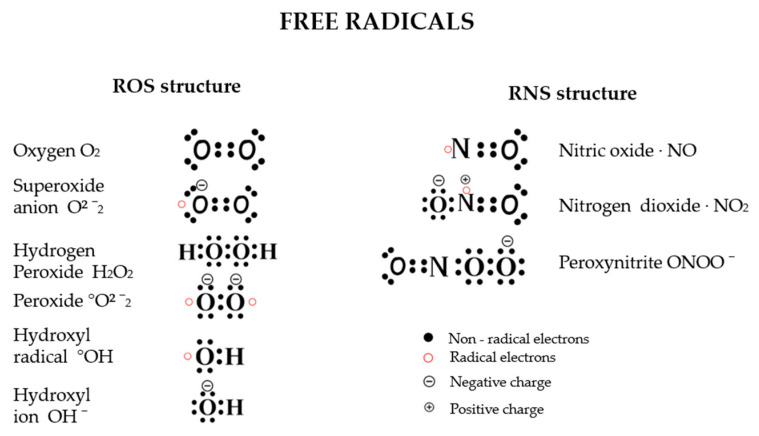
The structure of free radicals: Free radicals are highly reactive molecules with an unpaired electron count, capable of damaging cells. Reactive oxygen species (ROS), such as •OH, O_2_•^−^, •HO_2_, and H_2_O_2_, represent types of free radicals containing oxygen. Reactive nitrogen species (RNS) are highly active molecules derived from nitric oxide compounds, including •NO, ONOO^−^, and •NO_2_.

Free radicals are molecules that contain one or more unpaired electrons in their outer shell, causing them to be highly reactive and unstable. Free radicals are generally short-lived and often originate from oxygen and nitrogen [11]. These elements form highly reactive molecules known as reactive oxygen species (ROS) and reactive nitrogen species (RNS). ROS include superoxide anion radicals (O_2_^−^•), reactive hydroxyl radicals (OH•), hydroperoxyl radicals (HO_2_•), as well as other compounds like hydrogen peroxide (H_2_O_2_), hypochlorous acid (HOCl), and singlet oxygen (Figure 2) [12]. These molecules capture electrons from nearby molecules to stabilize themselves, which turns the nearby molecules into free radicals and initiates a chain reaction that can cause significant damage to cells and tissue. Free radicals can be generated in the body through enzymatic and non-enzymatic reactions (endogenous sources) or introduced from external sources (exogenous) such as pollution, smoking, and radiation [13]. Reactive oxygen species (ROS), including superoxide anions (O_2_^−^), hydroxyl radicals (OH^−^), and nitrogen-based radicals like nitric oxide (NO), are particularly harmful when their production exceeds the body’s antioxidant defenses [14]. Figure 2 demonstrates the structure of free radicals.

### 1.3. Oxidative Stress

**Figure 3:**
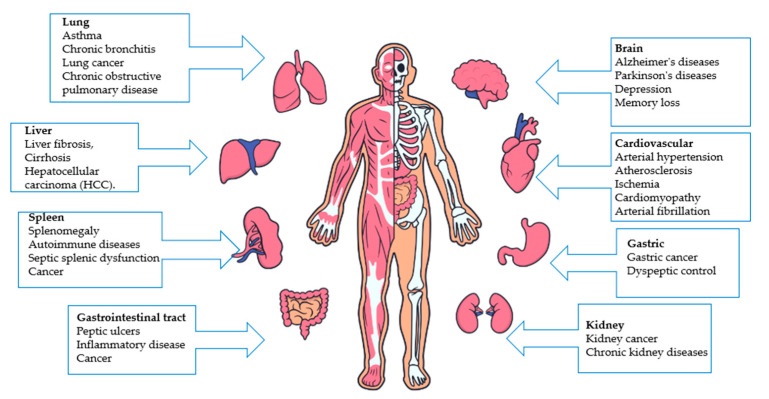
Schema illustrating human diseases caused by oxidative stress [17,18,19,20,21,22,23].

Oxidative stress is a form of biochemical imbalance where the formation of free radicals overwhelms the body’s antioxidant defense systems [15]. This imbalance damages cells and tissues by modifying essential biomolecules, including lipids, proteins, and nucleic acids. This oxidative stress is associated not only with xenobiotic toxicity but also with various conditions such as ischemia–reperfusion injury, vascular endothelium, deep injury, organ dysfunction, shock, inflammation, sepsis, diabetic retinopathy, cancer, cognitive impairment, cataract, pathophysiology and heart disease [16]. In addition, oxidative stress accelerates ageing by promoting tissue and organ dysfunction, highlighting its wide-ranging effects on health—Figure 3.

### 1.4. Oxidative Stress Markers

Reactive oxygen species (ROS) are compounds that are difficult to measure when assessing oxidative stress, primarily due to their short half-life, making them impractical as biomarkers [20]. However, ROS interacting with specific biological molecules leaves a unique chemical “fingerprint”. Biomarkers derived in this way can be used to evaluate oxidative damage or the effects of antioxidants, including therapeutic substances. ROS interacts with their surroundings in vivo as highly reactive substances, triggering and stimulating various endogenous mechanisms. They also react with many molecules, leaving fingerprints as reference points for specific assessments [24].

**Figure 4:**
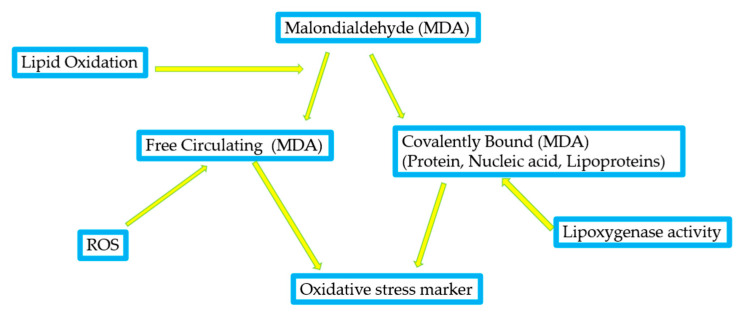
The involvement of reactive oxygen species (ROS) and lipoxygenase activity in lipid peroxidation and the formation of MDA involving free circulating MDA or covalently bound to proteins, nucleic acids, or lipoproteins during oxidative damage MDA is shown as a biomarker of oxidative stress linked with lipid oxidation [29,30].

Therefore, oxidative stress biomarkers are molecules that change in response to oxidative damage, allowing us to understand the level of stress and the effectiveness of antioxidant protection [25]. One example of an oxidative stress marker is malondialdehyde (MDA), a marker of lipid peroxidation. Lipids are sensitive to oxidation due to the double bonds in their molecular structure, which are reactive and less stable [26]. MDA can react with cellular components such as proteins, DNA, and lipids, causing cellular damage and dysfunction. The presence of MDA in cells and tissues is often used as an indicator of oxidative stress because it reflects the degree of lipid peroxidation and ROS-induced damage. Measurement of MDA levels can be used as a biomarker of oxidative stress and lipid peroxidation in clinical and experimental settings [27]. In addition, MDA can also be used as a therapeutic target to reduce the harmful effects of ROS, as antioxidants and other agents that reduce ROS production or scavenge free radicals can reduce MDA levels and reduce oxidative stress [28]. Figure 4 illustrates the involvement of reactive oxygen species (ROS) and lipoxygenase activity in lipid peroxidation, leading to the formation of malondialdehyde (MDA) as a biomarker of oxidative stress.

**Figure 5:**
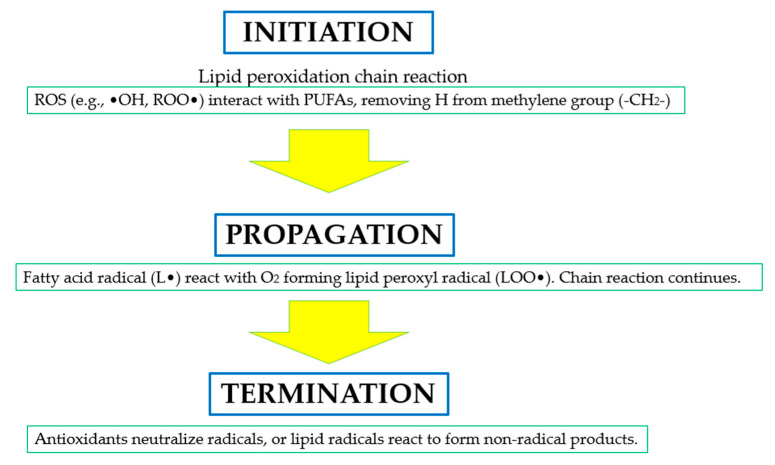
The three stages of lipid peroxidation, a critical oxidative process affecting polyunsaturated fatty acids (PUFAs) in biological membranes. During the initiation phase, reactive oxygen species (ROS), such as hydroxyl radicals (•OH) or peroxyl radicals (ROO•), abstract a hydrogen atom from the methylene group (-CH_2_-) in PUFAs, generating lipid radicals (L•). In the propagation phase, these lipid radicals react with molecular oxygen (O2), forming lipid peroxyl radicals (LOO•), propagating further chain reactions and amplifying oxidative damage. The termination phase involves neutralizing these radicals with antioxidants or forming non-radical products, halting the chain reaction [32,33,34].

Lipid peroxidation is a key process contributing to cellular damage during oxidative stress, significantly influencing the development of various diseases, including neurodegenerative and cardiovascular conditions [31]. Figure 5 illustrates the three stages of lipid peroxidation—initiation, propagation, and termination—and how ROS initiate chain reactions leading to lipid radicals, propagating oxidative damage until neutralized by antioxidants.

Other markers include glutathione S-transferase (GST), superoxide dismutase (SOD), catalase (CAT), glutathione peroxidase (GPx), and glutathione reductase (GR), as well as oxidized glutathione (GSSG) and reduced glutathione (GSH) [30,35,36]. During oxidative stress, (GST) levels may increase due to an activated antioxidant defense mechanism, as GST and reduced glutathione neutralize ROS [37]. An increase in (SOD) levels is also observed, which may be a compensatory mechanism by the body to protect itself from oxidative stress, as this enzyme is involved in breaking down superoxide anions. During oxidative stress, the activity of (CAT), as well as the levels of (GSH) and (GPx), diminish due to the insufficient availability of these enzymes to neutralize reactive oxygen species (ROS) effectively. Consequently, oxidized glutathione (GSSG) and glutathione reductase (GR) levels also decrease due to the intensified synthesis of ROS caused by oxidative stress [16,38].

**Figure 6:**
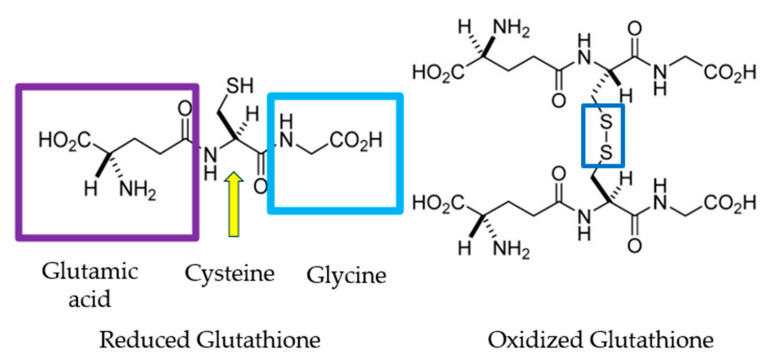
The figure illustrates the structural difference between reduced glutathione (GSH) and oxidized glutathione (GSSG). The reduced form (GSH) on the left consists of three amino acids: glutamic acid (highlighted by the purple square), cysteine, and glycine (highlighted by the blue square). The key feature is the thiol (–SH) group on the cysteine, which is marked by the yellow arrow and plays a crucial role in redox reactions. On the right, in the oxidized form (GSSG), two glutathione molecules are linked by a disulfide bond (S–S) between the sulfur atoms of the cysteine residues, indicated by the blue square. This bond replaces the free thiol groups in the reduced form, representing oxidative stress conditions.Glutathione exists in reduced glutathione (GSH) and oxidized glutathione (GSSG). The enzyme glutathione peroxidase catalyzes the conversion from GSH to GSSG, while the reverse process—from GSSG to GSH—is facilitated by glutathione reductase, using NADPH [38]. During oxidative stress, intracellular GSSG accumulates, leading to a decrease in the GSH/GSSG ratio. This ratio can indicate oxidative stress levels in tissues [40].

The (GSH) is a thiol compound, an endogenous intracellular antioxidant that ensues naturally in the body. It is a tripeptide comprising the well-known amino acids glycine, cysteine, and glutamic acid (Figure 6). One of its fundamental properties is its sensitivity to hydrogen peroxide due to the SH group in its structure, making it more reactive than catalase [39].

### 1.5. Antioxidants and Their Effects

Antioxidants have been and still constitute an exciting subject of investigation in the scientific world as their importance towards general human health and well-being has become apparent. Naturally occurring compounds, such as antioxidants, are crucial in eliminating free radicals, which would otherwise damage the cells and result in many other diseases, including cancer [41].

**Figure 7:**
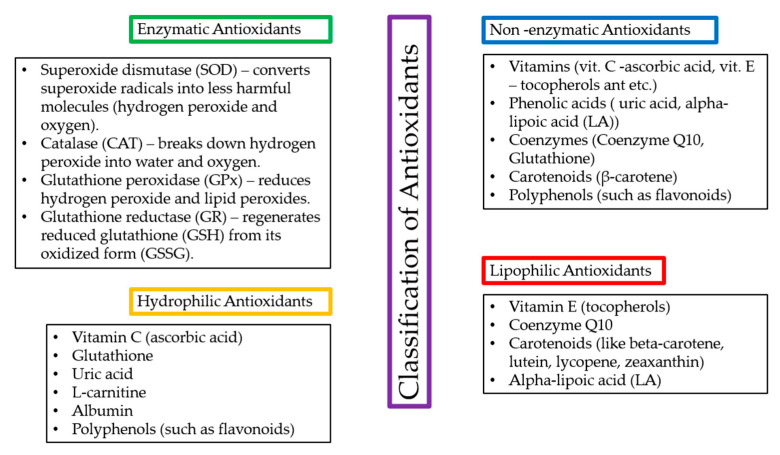
Classification of antioxidants with representative examples.

Antioxidants can be classified into enzymatic and non-enzymatic types. Enzymatic antioxidants function through biochemical reactions that neutralize (ROS), while non-enzymatic antioxidants directly scavenge free radicals or prevent their formation [42]. Also, their classification is based on their solubility factor in hydrophilic and lipophilic antioxidants. Hydrophilic antioxidants primarily act in the cell cytoplasm and blood plasma, while lipophilic antioxidants protect cell membranes by preventing lipid peroxidation [43,44]—Figure 7.

This study aims to evaluate the effects of grapefruit ethanolic extract (E) and naringin (NR) on biomarkers of antioxidant activity in the organs and blood of mice. It was hypothesized that the mechanisms by which E and NR reduce oxidative stress may differ. Grapefruit extract is expected to be more effective than naringin in reducing oxidative stress markers.

## 2. Results and Discussion

### 2.1. Quantitative Analysis of Phenolic Compounds and Selection of Extraction Sample

Before starting the in vivo study, the phenolic compounds naringin and naringenin were quantitatively analyzed on dried plant material using the HPLC method. The extraction process was carried out as described in the Materials and Methods Section 3.2, and the results are presented in Table 1.

The results demonstrated that ethanol concentration has a minor impact on the extraction efficiency. Instead, combining ultrasonic and reflux extraction plays a more significant role in achieving higher flavonoid yields. Similar findings were obtained from previous studies examining different parts of the citrus peel [45].

The UH70 sample had the highest naringin concentration (51.94 ± 2.6 mg/g), followed by UH50 (49.13 ± 2.46 mg/g). Both U50 and U70 samples yielded lower concentrations (42.04 ± 2.1 mg/g and 40.36 ± 2.02 mg/g, respectively), demonstrating the added benefit of combining ultrasonic and reflux extraction.

Similarly, the UH70 sample achieved the highest naringenin yield (64.22 ± 3.21 µg/g), with UH50 following closely (63.39 ± 3.17 µg/g).

Lower naringenin levels were found in U50 (56.81 ± 2.84 µg/g) and U70 (49.76 ± 2.49 µg/g), further showing the advantages of the combined extraction method. Meanwhile, UH70 provided slightly higher yields. However, its ethanol concentration (70%) required more extensive dilution to achieve 10% for in vivo studies, which may introduce variability.

The UH50 sample was selected for in vivo experiments due to its higher flavonoid content and ease of dilution, which allowed it to reach a 10% ethanol concentration for the in vivo studies. In the prepared 10% solution of the UH50 sample, the naringin concentration was calculated to be approximately 4.91 mg/mL and naringenin 6.339 μg/mL.

### 2.2. Result of GSH and MDA in Mouse Blood

**Figure 8:**
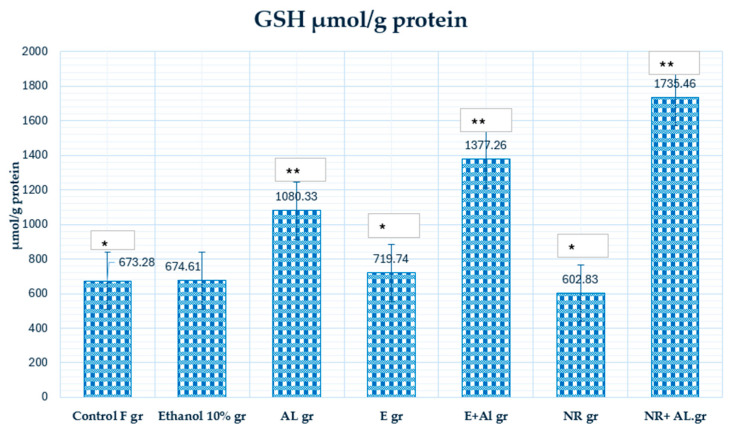
The concentration of GSH in mouse blood. * Indicates a statistically significant difference between the control group and the treated group with E and NR (*p* ≤ 0.05); ** indicates a statistically significant difference between the Al group and the treated group with the E + Al and NR + Al groups (*p* ≤ 0.05).

The changes in (GSH) levels observed in our study show the impact of (AlCl_3_) on oxidative stress and the protective effects of naringin and ethanol grapefruit extract treatments. The concentrations of GSH in the blood of control and experimental mice are given in Figure 8.

The reduced GSH levels in the NR-treated group (602.83 µmol/g) suggest that naringin was actively neutralize (ROS), resulting in depleted GSH reserves.

Conversely, the significant (*p* ≥ 005) increase in GSH levels in the NR + AlCl_3_ (1735.46 µmol/g protein) and E + AlCl_3_ (1377.26 µmol/g protein) groups highlights the protective effects of treatments. It can lead to their ability to stimulate GSH synthesis or moderate ROS levels, accordingly, preventing GSH depletion. Meanwhile, the higher GSH levels in the NR + AlCl_3_ group compared to the E + AlCl_3_ group suggest that naringin might substantially enhance the body’s natural antioxidant systems by activating GSH synthesis pathways.

NR and E demonstrated their efficacy in modulating GSH levels under oxidative stress conditions. However, GSH levels generally decrease during oxidative stress, so a compensatory mechanism is likely activated. Such observations are consistent with the results analyzed in the literature, highlighting the antioxidant properties of naringin and citrus peel extracts under oxidative stress conditions [46].

**Figure 9:**
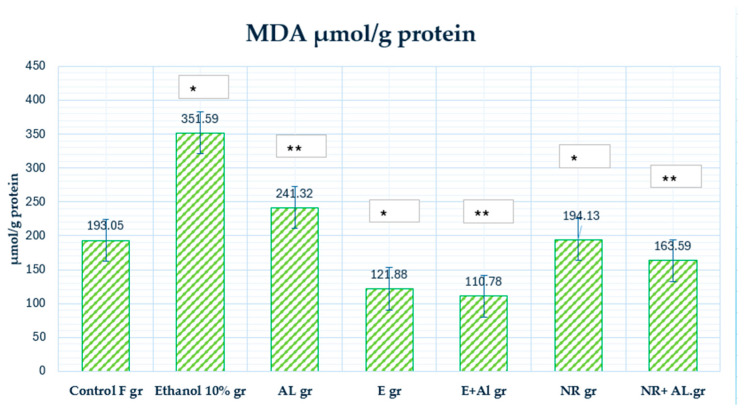
Demonstrates the concentration of MDA in mouse blood. * Indicates a statistically significant difference (*p* ≤ 0.05) between the ethanolic and treated groups; ** indicates a statistically significant difference (*p* ≤ 0.05) between the AlCl_3_ and treated groups.

The (MDA) is a biomarker that assesses lipid peroxidation and indicates oxidative stress. Increased MDA levels, as shown in the AlCl_3_ group, indicate increased oxidative stress, which can damage cell membranes, proteins, and DNA. This study observed changes in MDA concentration caused by exposure to AlCl_3_ and ethanol and the protective effect of treatment with naringin and extract (Figure 9).

MDA levels in the control group (193.05 µmol/g protein) indicate the initial oxidative state under physiological conditions.

Ethanol (351.59 µmol/g protein) significantly increased MDA levels compared to the control group, indicating ethanol’s pro-oxidative effect that enhances lipid peroxidation. This highlights the importance of controlling ethanol concentration in experimental designs to avoid misleading results.

The level of MDA in the AlCl_3_ group (241.32 µmol/g protein) was increased compared to the control group, indicating AlCl_3_-induced oxidative damage.

Treatment with the E alone (121.88 µmol/g protein) reduced MDA levels compared to the control group, indicating its antioxidant effect and ability to alleviate oxidative stress.

The observed decrease in MDA level (110.78 µmol/g protein) in the E + AlCl_3_ group indicates the ethanol extract’s protective effect against AlCl_3_-induced oxidative damage.

The level of MDA in the NR group (194.13 µmol/g of protein) was similar to that in the control group, but compared to the ethanol group, it showed a statistically significant decrease.

The average MDA level (163.59 µmol/g protein) decreased in the NR + AlCl_3_ group, but compared to the E + AlCl_3_ group, the extract’s effect on lipid peroxidation was higher. The results indicate that E substantially reduces lipid peroxidation more than NR, as evidenced by the lower MDA levels in the E + AlCl_3_ group.

### 2.3. Result of GSH, MDA and CAT in Mouse Brain

**Figure 10:**
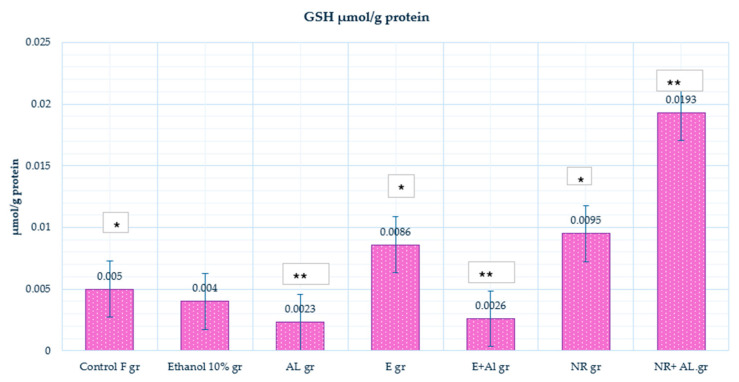
Glutathione reductase activity in the mouse brain. * Indicates a statistically significant difference (*p* ≤ 0.05) between the control and treated groups; ** indicates a statistically significant difference (*p* ≤ 0.05) between the AlCl_3_ and treated groups.

In the control group (0.005 μmol/g protein), GSH levels showed baseline antioxidant capacity in healthy brain tissues. Ethanol-treated mice (0.004 μmol/g protein) exhibited reduced GSH levels compared to the control, indicating ethanol-induced oxidative stress and depletion of cellular antioxidant reserves. The AlCl_3_ (0.0023 μmol/g protein) caused a higher decrease in GSH levels, confirming the severe oxidative stress caused by AlCl_3_, which disrupts redox homeostasis. The results are presented in Figure 10.

Treatment with the E alone (0.0086 μmol/g protein) significantly increased GSH levels compared to the control, indicating that the E enhances antioxidant capacity and promotes GSH synthesis under normal conditions. However, in the E + AlCl_3_ group (0.0026 μmol/g protein), GSH levels showed only a slight increase compared to AlCl_3_ alone, suggesting that the E provided limited protection against oxidative stress caused by AlCl_3_. In contrast, the NR-treated group (0.0095 μmol/g protein) significantly increased GSH levels, indicating its ability to enhance antioxidant capacity under normal conditions. Meanwhile, in the NR + AlCl_3_ group (0.0193 μmol/g protein), GSH levels were significantly restored, reaching the control levels, suggesting that NR neutralized oxidative stress and strongly promoted GSH synthesis.

**Figure 11:**
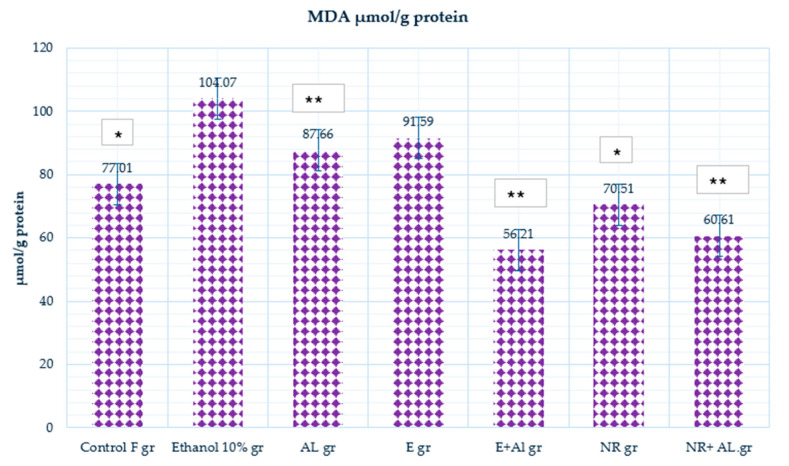
MDA activity in the mouse brain. * Indicates a statistically significant difference (*p* ≤ 0.05) between the control and treated groups; ** indicates a statistically significant difference (*p* ≤ 0.05) between the AlCl_3_ and treated groups.

MDA, a marker of lipid peroxidation, reflects the extent of oxidative damage. In the control group (77.01 μmol/g protein), MDA levels represent baseline oxidative conditions with minimal lipid peroxidation. The ethanol-treated group (104.07 μmol/g protein) increased MDA levels, highlighting ethanol’s role in generating ROS and promoting lipid peroxidation. Similarly, the AlCl_3_ group increase (87.66 μmol/g protein) MDA level in mice brains indicated oxidative stress. The results are presented in Figure 11.

The combination of the E and AlCl_3_ (56.21 μmol/g protein) significantly lowered MDA levels, showing that the E effectively neutralized oxidative stress caused by AlCl_3_. The NR-treated group (70.51 μmol/g protein) showed reduced MDA levels compared to the control, reflecting naringin’s preventive role against oxidative damage. In the NR + AlCl_3_ group (60.61 μmol/g protein), naringin reduced MDA levels, demonstrating its strong ability to mitigate lipid peroxidation and oxidative damage under AlCl_3_ exposure. In this case, E has a significantly more potent effect on the MDA level than NR.

**Figure 12:**
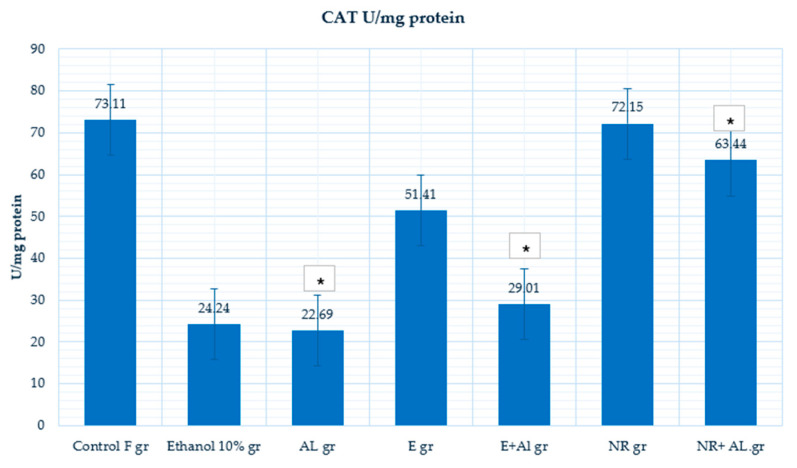
CAT activity in the mouse brain. * Indicates a statistically significant difference (*p* ≤ 0.05) between the AlCl_3_ and treated groups.

CAT is an essential antioxidant enzyme detoxing hydrogen peroxide, protecting cells from oxidative damage. The control group (73.11 U/mg protein) indicates regular CAT activity, reflecting strong enzymatic antioxidant defenses. CAT activity decreased significantly in the ethanol-treated group (24.24 U/mg protein), indicating that ethanol impairs enzymatic antioxidant defenses. Similarly, AlCl_3_ (22.69 U/mg protein) reduced CAT activity, confirming that AlCl_3_ suppresses antioxidant enzymes to increase oxidative stress. The results are presented in Figure 12.

However, in the E + AlCl_3_ group (29.01 U/mg protein), CAT activity showed only a slight increase, indicating limited protection against AlCl_3_-induced oxidative stress, but did not reach a control group. The NR-treated group (72.15 U/mg protein) maintained CAT activity at near control levels, highlighting its ability to sustain enzymatic antioxidant defenses under normal conditions. In the NR + AlCl_3_ group (63.44 U/mg protein), CAT activity was significantly restored, compared with the Al group, but did not reach the control group (73.11 U/mg protein).

The E showed a stronger ability to reduce MDA levels, demonstrating its effectiveness in preventing lipid peroxidation under the oxidative stress caused by AlCl_3_. Meanwhile, NR significantly increases GSH levels and restores CAT activity.

### 2.4. Result of GSH, MDA and CAT in Mouse Liver

**Figure 13:**
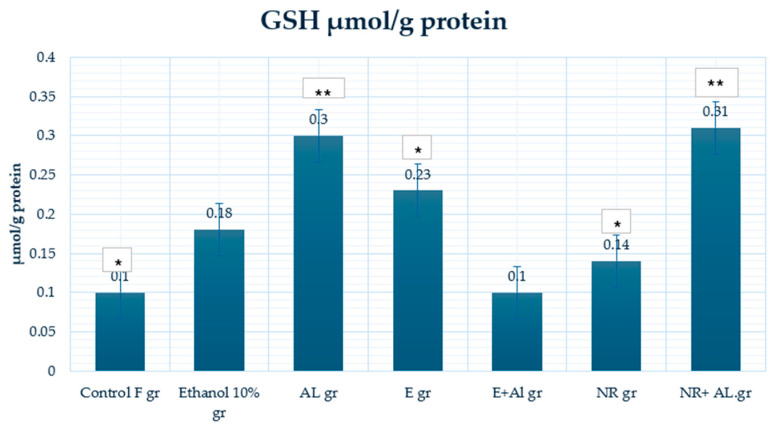
GSH activity in the mouse liver. * Indicates a statistically significant difference (*p* ≤ 0.05) between the control and treated groups; ** indicates a statistically significant difference (*p* ≤ 0.05) between the AlCl_3_ and treated groups.

**Figure 14:**
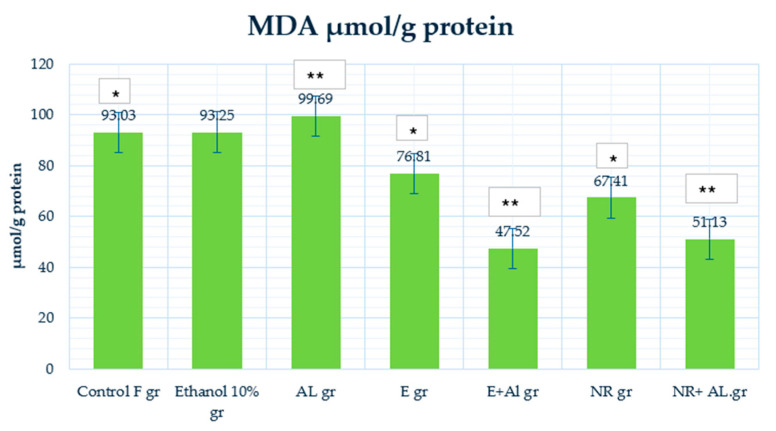
MDA activity in the mouse liver. * Indicates a statistically significant difference (*p* ≤ 0.05) between the control and treated groups; ** indicates a statistically significant difference (*p* ≤ 0.05) between the AlCl_3_ and treated groups.

**Figure 15:**
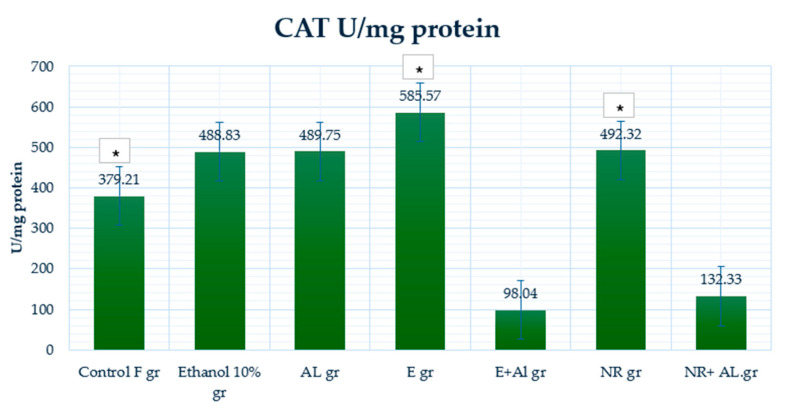
CAT activity in the mouse liver. * Indicates a statistically significant difference (*p* ≤ 0.05) between the ethanolic and treated groups.

The effects of treatments on reduced (GSH), (MDA), and (CAT) activity in the liver are shown in Figure 13, Figure 14 and Figure 15. During cervical dissection, notable physical changes were observed in the livers of AlCl_3_-treated mice. These livers appeared enlarged, had irregular edges, and, in some cases, adhered to surrounding organs, indicating pathological changes likely caused by AlCl_3_-induced oxidative stress and toxicity.

In the control group (0.1 μmol/g protein), baseline GSH levels show standard antioxidant capacity. The ethanol-treated group (0.18 μmol/g protein) showed a slight increase in GSH, likely due to a compensatory response to oxidative stress caused by ethanol metabolism. The AlCl_3_ group have the highest GSH levels (0.3 μmol/g protein), possibly reflecting an acute cellular response to counteract AlCl_3_-induced oxidative stress.

The E group (0.23 μmol/g protein) increases on average GSH levels, suggesting its role in enhancing antioxidant defenses under normal conditions. However, in the extract (E) + AlCl_3_ group (0.1 μmol/g protein), GSH levels dropped to control levels, indicating that the E provided limited protection against AlCl_3_-induced oxidative stress in the liver. In contrast, the NR group (0.14 μmol/g protein) slightly increased GSH levels compared to the control. In comparison, the NR + AlCl_3_ group (0.31 μmol/g protein) significantly restored GSH levels, indicating NR capacity to stimulate GSH synthesis and neutralize AlCl_3_ toxicity.

The MDA control group (93.03 μmol/g protein) demonstrated baseline lipid peroxidation levels. The ethanol-treated group (93.25 μmol/g protein) showed similar MDA levels, indicating that ethanol did not significantly increase lipid peroxidation in the liver. However, the AlCl_3_ group (99.69 μmol/g protein) increase MDA levels, confirming AlCl_3_-induced oxidative stress and membrane lipid damage.

Treatment with the E alone (76.81 μmol/g protein) significantly reduced MDA levels, demonstrating its protective effect in lipid peroxidation under normal conditions. The E + AlCl_3_ group (47.52 μmol/g protein) decreases MDA levels compared to the AlCl_3_ group, highlighting the E strong antioxidative properties against AlCl_3_-induced lipid damage. Similarly, the NR group (67.41 μmol/g protein) reduced MDA levels compared to the control, indicating its preventive effect on oxidative damage. The NR + AlCl_3_ group (51.13 μmol/g protein) decreased MDA levels, demonstrating that NR effectively counteracts AlCl_3_-induced lipid peroxidation.

The control group (379.21 U/mg protein) displayed baseline CAT activity and enzymatic defense mechanisms. The ethanol-treated group (488.83 μmol/g protein) and AlCl_3_ group (489.75 U/mg protein) showed higher CAT activity, likely reflecting an adaptive response to increased ROS production.

The E group (585.57 U/mg protein) demonstrates the highest CAT activity, suggesting that the E strongly enhances enzymatic antioxidant defenses under normal conditions. However, in the E + AlCl_3_ group (98.04 U/mg protein), CAT activity dropped significantly compared to the AlCl_3_ group, indicating that the E had limited efficacy in restoring CAT activity under oxidative stress. The NR group (492.32 U/mg protein) maintained CAT activity, demonstrating NR’s ability to support enzymatic defenses under normal conditions. In the NR + AlCl_3_ group (132.33 U/mg protein), CAT activity improved significantly compared to the E + AlCl_3_ group, indicating that NR provided better protection and restoration of enzymatic activity under oxidative stress.

Our findings on the effects of NR and E on oxidative stress markers GSH, MDA, and CAT are in close agreement with other studies using plant-derived antioxidants. These comparisons demonstrate natural extracts’ unique properties and mechanisms in reducing oxidative stress.

M. Papaefthimiou et al. demonstrated that Stevia leaf extracts significantly reduced MDA levels while restoring GSH concentrations and antioxidant enzyme activity in experimental rat models [4]. It is similar to our findings that E effectively reduced MDA levels in liver and brain tissues, demonstrating its potential to limit lipid peroxidation under oxidative stress

G. M. Iova et al. found that curcumin and rutin restored GSH and reduced MDA levels in rats with experimentally induced oxidative stress [3]. Similarly, in our study, NR restored GSH levels, particularly in the brain and liver, highlighting its strong ability to counteract oxidative damage.

The findings from the *Origanum onites* L. study align closely with our research, as both demonstrate the effectiveness of plant-based antioxidants in reducing oxidative stress. In the *O. onites* study, the essential oil effectively reduced MDA levels, similar to the firm lipid peroxidation control observed with E in our study. Meanwhile, *O. onites* extract remarkably restored GSH levels and enhanced enzymatic protection, which parallels the effects of NR in our findings. Both studies highlight these antioxidants’ complementary and tissue-specific roles in protecting against oxidative damage [47].

Sadauskienė et al. highlighted the ability of natural extracts to enhance GSH levels and restore CAT activity in liver and brain tissues under oxidative stress conditions [48]. It is consistent with our results, where NR effectively restored GSH and CAT activity, especially in the brain and liver, indicating its broad-spectrum antioxidant capacity.

The results of our study are strongly aligned with findings from another plant-based antioxidant research. While ethanolic grapefruit extract reduced lipid peroxidation (MDA) in the liver and brain, naringin showed broader antioxidant effects by restoring GSH levels and CAT activity. Future studies could explore their synergistic application to optimize therapeutic strategies related to oxidative stress.

## 3. Materials and Methods

### 3.1. Material

Naringin (NR) (≥95% purity)—Sigma Aldrich (Steinheim, Germany); ethanol (96%)—Vilniaus Degtinė (Vilnius, Lithuania); AlCl_3_ (aluminum chloride)—Sigma Aldrich (Steinheim, Germany); GSH (reduced glutathione)—Sigma Aldrich (Steinheim, Germany); MDA (malondialdehyde)—Sigma Aldrich (Steinheim, Germany); TBA (thiobarbituric acid), tris-HCl (tris-hydrochloride), DTNB (5,5-dithiobis-(2-nitrobenzoic acid) were supplied by Serva, Heidelberg, Germany; TCA (trichloroacetic acid)—Merck (Darmstadt, Germany).

Analytical balance—Kern ABT 120-4M (Kern & Sohn GmbH, Balingen, Germany) max 120 g, min 10 mg, e = 1 mg, d = 0.1 mg; ultrasonic bath—Grant Instruments™ XUB12 Digital Bath (Grant Instruments Ltd., Shepreth, UK); Memmert UN 55 Laboratory Oven (Memmert GmbH + Co. KG, Büchenbach, Germany); centrifuge Sigma 3-18K, (Sigma Laborzentrifugen GmbH, Osterode am Harz, Germany); HPLC System—Waters 2695 Liquid Chromatography (Waters Corporation, Milford, MA, USA) with photodiode array detector (Waters 996, wavelength range 200–400 nm); chromatographic column—ACE C18 (250 mm × 4.6 mm, 5 μm particle size) (Advanced Chromatography Technologies Ltd., Aberdeen, UK); spectrophotometer—PerkinElmer Lambda 25 UV/Vis Spectrometer (PerkinElmer, Shelton, CT, USA).

### 3.2. Preparation of Naringin Solution for In Vivo Studies

Based on the scientific literature, a 500 mg/kg naringin concentration was selected to evaluate its antioxidant effects in an AlCl_3_-induced oxidative stress mouse model. This dosage was chosen based on previous in vivo studies demonstrating its efficacy in mitigating oxidative stress and restoring antioxidant defense biomarkers [49,50]. To prepare 100 mL of solution, 5 g of naringin powder was dissolved in a 10% ethanol solution. The low ethanol concentration (10%) was chosen to minimize potential effects on the mice. The final naringin solution concentration was 50 mg/mL, and it was stored at ±4 °C until use [51,52].

### 3.3. Preparation and Extraction of Flavonoids from Citrus x paradisi L. Peels

**Figure 16:**
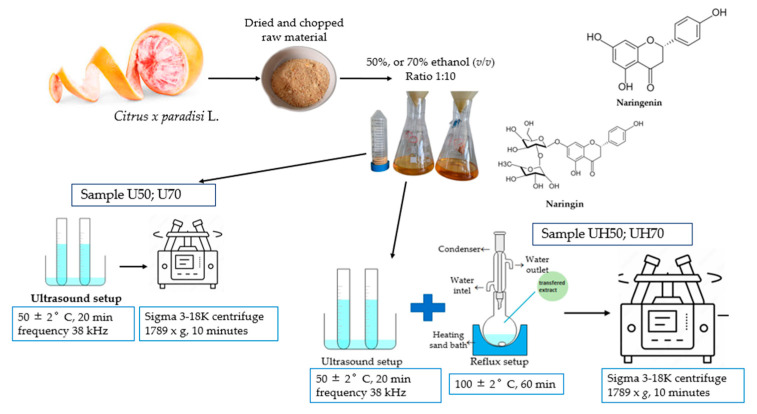
The sample ID represents the extraction method and ethanol concentration used. U indicates ultrasonic extraction, while UH combines ultrasonic and reflux extraction—the numbers correspond to ethanol concentrations: 50 for 50% ethanol and 70 for 70% ethanol.

*Citrus x paradisi* L. peels, a commonly discarded plant material, were dried at 60 °C for 8 h in the Department of Drug Technology and Social Pharmacy, Lithuanian University of Health Sciences, using a Memmert UN 55 Laboratory Oven (Memmert GmbH + Co. KG, Büchenbach, Germany). The dried peels were then ground to a fine powder. Flavonoid extraction was conducted following methodologies shown in previous studies, with a detailed schematic of the process presented in Figure 16 [45,53].

For the extraction, ethanol solutions at concentrations of 50% and 70% (*v*/*v*) were used with a ratio of 1:10. Two extraction processes were evaluated. In the first process, ultrasonic extraction was performed using a Grant Instruments™ XUB12 Digital bath (Grant Instruments Ltd., Shepreth, UK), operating at 38 kHz for 20 min under controlled conditions of 50 ± 2 °C. The second step involved transferring the ultrasonically treated mixture to a reflux system for an additional 60 min extraction at 100 ± 2 °C temperature.

The extract was allowed to cool to room temperature and centrifuged with Sigma 3-18K centrifuge at room temperature (25 ± 5 °C) (Sigma Laborzentrifugen GmbH, Osterode am Harz, Germany) at 1789× *g* for 10 min to separate the solid residue from the liquid extract. The supernatant was filtered through a 0.22 μm PVDF membrane to remove residual particles. The samples were further analyzed, and their quantities were evaluated using HPLC.

### 3.4. Chromatographic Analysis of Citrus x paradisi L. Peels Ethanolic Extract: HLPC Analysis for Phenolic Compounds

A Waters 2695 liquid chromatography with a photodiode array detector (Waters 996, 200–400 nm wavelength range) was used in this study. In addition, a chromatographic column ACE C18 (250 mm × 4.6 mm) (Advanced Chromatography Technologies Ltd., Aberdeen, UK) with a sorbent particle size of 5 μm was used to separate the biologically active compounds. The following are the details of the procedure for the HPLC method. The HPLC analysis was performed to separate and quantify biologically active compounds in the ethanolic extract of *Citrus x paradisi* L. peels, including naringin and naringenin. The gradient elution method was applied to these extracts to achieve optimal separation of the target flavonoids. Then, 10 μL of each extract was injected and analyzed at 280 nm. Eluent A: acetonitrile; eluent B: water at a 1 mL/min rate. Gradient elution:10% of A from 0 to 5 min, from 5 to 25 min 20%, from 25 to 30 min 40%, from 30 to 35 min 100%, 35 min 100%, 36 min 10%. The temperature of the column was 25 °C. The peaks were identified by comparing their UV–vis spectra and retention times to those of authentic reference standards. Each extract was analyzed in duplicate as a technical repetition to confirm the reproducibility of the HPLC method. Naringin and naringenin were used as reference standards for calibration, retention time identification, and quantification validation.

**Figure 17:**
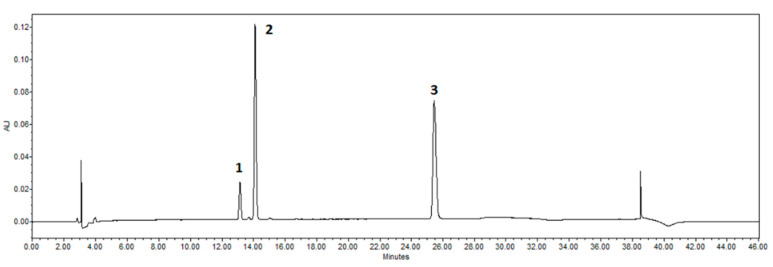
Chromatograms of standards detected by HPLC. Peaks identified: 1—narirutin, 2—naringin, 3—naringenin.

The quantification and validation followed the methodical revision of natural products presented by Wolfender (2009) [54]. Standard stock solutions of 100 μg/mL primary concentrations for naringin and naringenin were prepared in 70% methanol, and calibration curves were constructed using 6 different standard solution concentrations (Naringin: 1.166, 3.499, 8.332, 16.666, 25.000, and 33.343 μg/mL; Naringenin: 0.472, 1.889, 3.774, 7.547, 11.386, and 15.125 μg/mL). Three injections per concentration were performed to determine linearity. Naringin and naringenin were plotted against the known concentrations of their associated standard solutions to establish calibration equations. A linear regression equation was calculated using the least-squares method. The regression coefficients of all calibration curves were R^2^ > 0.999, confirming the linearity of the concentration ranges. The method sensitivity was evaluated by determining the limit of detection (LOD)and quantitation (LOQ) Table 2. LOD and LOQ were calculated as the concentrations that gave signal-to-noise ratios of 3 to 10, respectively. A standard mixture of naringin and naringenin was used for intra-day and inter-day precision testing. The method precision was demonstrated by performing five replication-consecutive injections of the usual mix on the same day on four different days. The results are reported in terms of RSD. This study analyzed standards (naringin and naringenin), and their retention time and spectra were compared with the prepared extracts. The linearity was determined by estimating the correlation coefficient ‘R^2^’ of the calibration curve (Figure 17) (naringin ‘R^2^’ = 0.999923, naringenin ‘R^2^’ = 0.999924), and the peak areas were used for quantification, Table 2. The linearity range of naringin was 1.166 to 33.343 μg/mL, and naringenin was 0.472 to 15.125 μg/mL. The results were expressed as μg/g and mg/g dry weight (DW) of naringenin and naringin, respectively.

### 3.5. Animal Model

Experiments were performed with 4- to 6-week-old Balb C white laboratory mice weighing 20–25 g. All experiments were performed according to the Republic of Lithuania Law on the Care, Keeping, and Use of Animals (License of State Veterinary Service for Working with Laboratory Animals Nr. G2-275). Although this study observed ethical guidelines for animal research, further studies are needed to confirm the findings of clinical trials in humans, given species-specific differences in oxidative stress responses.

The mice were housed under standard laboratory conditions, maintained at a temperature of 22 ± 2 °C, with 55 ± 5% relative humidity, and a 12-h light-dark cycle. They had constant access to feed and water throughout the experiment. The diet consisted of Ab “Kauno Grūdai” Visaverčiai pašarai triušiams KG NATURE, a GMO-free complete feed formulated to provide balanced nutrition. The animals were supplied with filtered tap water, free from chemical additives.

**Figure 18:**
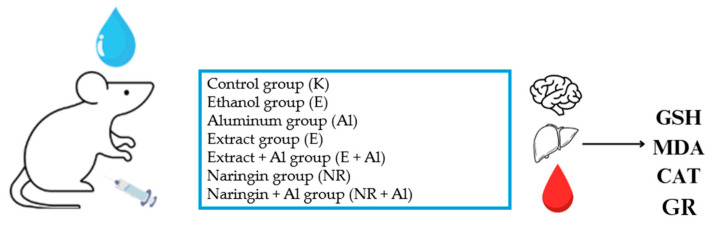
Experimental design and biomarkers assessed: reduced glutathione (GSH), malondialdehyde (MDA), and catalase (CAT), glutathione reductase (GR).

This study involved 7 groups housed in separate cages containing 5 mice per group. The ethanolic *Citrus x paradisi* L. peel extract, and naringin solution (50 mg/mL body weight) was administered intragastrically for 21 days. The experimental design is illustrated in Figure 18.

The control 1 group received NaCl 0.9% (the saline solution) for 21 days.

The control 2 group received 10% ethanol solution for 21 days.

The control 3 group AlCl_3_ group (dissolved in saline) was injected intraperitoneally at 7.5 mg of Al^3+^/kg body weight (0.15 LD50) in the AlCl_3_ for 21 days.

The 4th group received ethanolic *Citrus x paradisi* L. peel extract intragastrical for 21 days.

The 5th group received ethanolic naringin 50 mg/mL solution intragastrical for 21 days.

The 6th group in which AlCl_3_ (7.5 mg Al^3+^/kg body weight) was administered intraperitoneally, followed by an intragastric administration of naringin solution (50 mg/mL) after a 20 min interval for 21 days.

The 7th group was treated with AlCl_3_ intraperitoneally at the exact dosage, followed by intragastric administration of ethanolic *Citrus x paradisi* L. extract after a 20 min interval for 21 days.

This study involves collecting data on the body weight of mice in each group and monitoring changes in body mass continuously over 21 days. AlCl_3_ solution was selected to induce oxidative stress due to its ability to indirectly promote ROS (reactive oxygen species) production via the Fenton reaction [55]. This reaction generates highly reactive hydroxyl radicals. The AlCl_3_ solution was prepared by dissolving AlCl_3_ in 0.9% sodium chloride. The concentration of the Al solution for the mice was determined based on scientific literature and calibrated according to the median lethal dose (LD50) of Al, which is 7.5 mg (0.15 LD50) per kilogram of body weight [56].

The AlCl_3_ solution was administered to the mice intraperitoneally using a 1 mL insulin syringe. The ethanolic *Citrus x paradisi* L. peel extracts and naringin solution were delivered orally to the stomach using a specialized 1 mL syringe equipped with a probe designed for laboratory mice. The dosage of the test solutions was adjusted according to the body weight of the mice and any changes in their weight observed throughout this study.

After the 21-day study period, laboratory mice were weighed and euthanized via cervical dislocation followed by decapitation. This procedure complied with the European Convention for the Protection of Vertebrate Animals used for Experimental and Other Scientific Purposes [57]. Blood samples were collected into heparin-treated tubes to prevent coagulation. Blood analyses were performed immediately on the same day to ensure reliable results.

The organs of the laboratory mice, including the liver and brain, were carefully prepared for further analysis. They were rinsed with physiological saline to remove residual blood and placed in Petri dishes. The organs were frozen at −40 °C to preserve their integrity until subsequent experiments. Weighed organs were homogenized in 9 volumes of cold 1.15% KCl solution relative to the organ weight, resulting in a 10% homogenate. The homogenate was then centrifuged at 15,000× *g* for 15 min.

### 3.6. Determination of GSH Concentration in Laboratory Mice Blood

The reduced glutathione (GSH) concentration in laboratory mice blood was determined using the method described by Sedlak and Lindsay [58]. This method relies on a reduction reaction in which GSH reacts with Ellman’s reagent DTNG (5,5′-dithiobis-(2-nitrobenzoic acid). GSH and DTNB form a yellow-colored complex in an alkaline medium during this reaction.

To determine GSH concentration, 200 µL of blood was mixed with 1.8 mL deionized water and 2 mL of 0.6 M HClO_4_. The prepared mixture was centrifuged at 3000 rpm for 10 min. After centrifugation, 1 mL of the supernatant was mixed with 3 mL of 0.4 M Tris–HCl buffer solution (pH 9.2) and 50 µL of Ellman’s reagent solution. The same procedure was followed to prepare the control solution, except that 1 mL of deionized water was used instead of the supernatant. The color intensity of the reaction mixture was measured spectrophotometrically at a wavelength of 412 nm. The GSH concentration in the blood was expressed in μmol/L and calculated using the appropriate formula [59].

(1) $$Cµ\frac{mol}{L}=A\times 1488.9705\times V_{0}$$

C—GSH concentration in μmol/L; A—absorbance of the supernatant at 412 nm wavelength; 1488.9705—coefficient; V_0_—volume of the supernatant in ml.

### 3.7. Determination of MDA Concentration in Laboratory Mice Blood

The concentration of MDA in laboratory mice blood was determined using the method described by Seliutina and Selutin, which is based on the reaction between MDA and TBR (thiobarbituric acid) [60]. During the reaction, MDA reacts with TBR to form MDA-TBR_2_ complexes, which exhibit a pink color and quantify MDA concentration in erythrocytes [47].

For the determination of MDA concentration, both test and control mixtures were prepared. The test mixtures comprised 2 mL of deionized water, 100 µL of mouse blood, 1 mL of 10% trichloroacetic acid, and 2 mL of 0.5% thiobarbituric acid solution. 2 mL of deionized water was used instead of the 0.5% TBR solution for the control mixtures. The mixtures were stirred with a glass rod and incubated in a water bath for 30 min. After incubation, the samples were cooled in an ice bath.

Once cooled, the samples were centrifuged at 3000 rpm for 15 min. The upper, transparent layer of the centrifuged mixture was analyzed using a spectrophotometer to measure absorbance at a wavelength of 540 nm. The MDA concentration in mice blood was calculated using the following formula, with results expressed in µmol/L:

(2) c=E×1250

where c MDA concentration in the blood (µmol/L); E—absorbance of the test sample at 540 nm; 1250—coefficient.

### 3.8. Determination of GSH Concentration in Laboratory Mouse Organs

The concentration of GSH was measured following the method outlined by Sadauskiene et al. [48,61]. Mouse liver or brain tissues were homogenized in 5% trichloroacetic acid at a ratio of six volumes of the solution to one of tissue weight. The homogenate was then centrifuged at 10,000× *g* for 7 min.

The supernatant was reacted with DTNB (Ellman’s reagent, 5,5-dithiobis (2-nitrobenzoic acid)). Each reaction mixture (3 mL) was prepared by combining 2 mL of 0.6 mM DTNB in 0.2 M sodium phosphate buffer (pH 8.0), 0.2 mL of the supernatant, and 0.8 mL of 0.2 M phosphate buffer. The resulting compound exhibited maximum light absorption at 412 nm. GSH content was calculated and expressed as µmol/g of fresh tissue weight.

(3) $$C\mu mol/g=\frac{A\times 3\times V_{gal}}{2.72\times w}$$

C—GSH concentration in tissues (µmol/g); A—absorbance value of the test sample; 3—coefficient; V_gal_—volume of the supernatant; 2.72—coefficient; w—tissue weight.

### 3.9. GR Activity Assay

The method for determining glutathione reductase (GR) activity is based on measuring the decrease in absorbance at 340 nm due to NADP^+^ oxidation during the reduction in oxidized glutathione (GSSG) catalyzed by GR.

The reaction mixture (2 mL, excluding sample volume) contains 0.05 M phosphate buffer (pH 7.8), 1 mM EDTA, 0.16 mM NADP^+^, and 0.8 mM oxidized glutathione. The reaction is initiated by adding 20 µL of the sample, and the absorbance is measured at 340 nm at 0 min (E_0_) and 3.5 min (E_a_) [62].

GR activity is calculated using the formula:

(4) $$A=\frac{\left(E_{0}-E_{a}\right)}{\left(T\times V_{total}\times P\right)}\times \frac{1}{6.22\times C}$$

T—reaction time (min), V_total_ is cuvette volume (2 mL), P—dilution factor, 6.22—extinction coefficient for glutathione (cm^−1^ mM^−1^), and C—protein concentration (mg/mL).

### 3.10. Determination of MDA Concentration in Laboratory Mouse Organs

The final product of lipid peroxidation, (MDA), reacts with TBA (thiobarbituric acid) to form a colored complex, which can be quantified spectrophotometrically. The results are expressed as µmol/g of wet tissue weight [61]. Brain or liver tissues were excised and homogenized in a 9-fold volume (relative to tissue weight) of cold 1.15% potassium chloride (KCl) solution to produce a 10% homogenate.

To 0.5 mL of the homogenate, 3 mL of 1% phosphoric acid (H_3_PO_4_) and 1 mL of 0.6% TBA solution were added. The mixture was heated in a boiling water bath for 45 min to facilitate the reaction. After cooling, 4 mL of n-butanol was added to the sample, and the mixture was thoroughly mixed. The butanol phase was separated by centrifugation, and the absorbance of the supernatant was measured spectrophotometrically at 535 nm and 520 nm. This method enables accurate quantification of MDA as formula.

(5) $$Cµmol/g=\frac{∆O.V.\times 100,000}{133}$$

C—MDA concentration in tissues (µmol/g); ΔO.V.—difference in absorbance at 520 nm and 535 nm wavelengths; 100,000 and 133—coefficients.

### 3.11. (CAT) Activity Assay

Catalase activity in brain and liver homogenates was measured using the method described by Sadauskiene et al. [62]. This assay relies on the catalase decomposition of hydrogen peroxide (H_2_O_2_). The reaction mixture consisted of 50 mM Tris-HCl buffer (pH 7.4) containing 18 mM H_2_O_2_ (buffer-substrate mixture) and 100 μL of tissue homogenate. The mixture was incubated at 37 °C for 180 s to allow the enzymatic reaction to occur.

The reaction was terminated by adding 2.0 mL of 4.5% ammonium molybdate, which forms a yellow complex with residual hydrogen peroxide. The absorbance of this complex was measured at 410 nm using a spectrophotometer (PerkinElmer Lambda 25 UV/Vis Spectrometer (PerkinElmer, Shelton, CT, USA). A blank control was prepared by incubating the buffer-substrate mixture for 180 s, adding ammonium molybdate and 100 μL of the tissue homogenate.

The catalase activity was expressed as units per mg of protein (U/mg protein). One unit of CAT (U) corresponds to the decomposition of 1 μmol of H_2_O_2_ per minute under the assay conditions. This method provides a reliable measure of CAT activity in tissue samples.

(6) $$A=\frac{(E_{K}-E_{B})\times 12\times 10^{3}\times 4.1\times 10^{6}}{22.2\times 10^{6}\times t}$$

*A*—CAT activity (U/mL); *E_K_*—mean absorbance of the control sample at 410 nm wavelength; *E_B_*—mean absorbance of the test sample at 410 nm wavelength; 12 × 10^3^—dilution factor; 4.1 × 10^6^—conversion coefficient to µmol; 22.2 × 10^6^—molar extinction coefficient of H_2_O_2_; *t*—incubation time (3 min).

### 3.12. Statistical Analysis

The statistical analysis evaluated the effects of naringin, and grapefruit extract on oxidative stress markers (GSH, MDA, and CAT) in mice’s blood, liver, and brain, comparing the treatment groups to control groups. Data were expressed as the mean ± SD (standard error of the mean). Statistical significance was determined using one-way analysis of difference (ANOVA) and the unpaired Student *t*-test. The value of *p* < 0.05 was considered statistically significant (SPSS version 20.0, IBM, Armonk, NY, USA).

## 4. Conclusions

This study demonstrates the effectiveness of combining ultrasound and reflux extraction (UH) methods (as example by the UH50 sample) to achieve higher yields of naringin and naringenin from *Citrus x paradisi* L. Our in vivo findings show different mechanisms through which grapefruit extract and naringin mitigate oxidative stress E significantly reduced lipid peroxidation, as evidenced by a marked decrease in (MDA) levels in the blood (Al group 241.32 µmol/g protein, E + AL group 110.78 µmol/g protein), respectively; in the liver (Al group 76.81 µmol/g protein, E + AL group 47.52 µmol/g protein), respectively; in the brain (Al group 87.66 µmol/g protein, E + AL group 56.21 µmol/g protein), respectively. Meanwhile, NR significantly impacted the antioxidant defense system by restoring (GSH) levels: in the blood (Al group 1080.33 µmol/g protein, E + AL group 1735.46 µmol/g protein), respectively; in the brain (Al group 0.0023 µmol/g protein, E + AL group 0.0193 µmol/g protein) respectively; in the liver (Al group 0.3 µmol/g protein, E + AL group 0.31 µmol/g protein) respectively, and (CAT) activity: in the brain (Al group 23.69 U/mg protein, E + AL group 63.44 U/mg protein) respectively; in the liver (Al group 489.75 U/mg protein, E + AL group 132.33 U/mg protein) respectively.

The hypothesis that E surpasses NR in reducing oxidative stress markers was only partially confirmed. However, E demonstrated excellent control over lipid oxidative damage, and NR proved more effective in restoring key antioxidant biomarkers, particularly under aluminum chloride (AlCl_3_)-induced oxidative stress. These findings suggest that combining E and NR may offer synergistic benefits, improving protection against oxidative damage through complementary pathways.

This research highlights the potential of grapefruit-derived bioactive compounds and lays the foundation for exploring combined therapeutic strategies to manage oxidative stress in future studies.

## Figures and Tables

**Table 1:** Quantification of naringin and naringenin in *Citrus x paradisi* L. peel extracts using different extraction methods and ethanol concentrations. The sample ID represents the extraction method and ethanol concentration used. U—ultrasonic extraction, UH—combines ultrasonic and reflux extraction—the numbers correspond to ethanol concentrations: 50 for 50% ethanol and 70 for 70% ethanol.

Sample ID	Naringin (mg/g)	Naringenin (µg/g)
U50	42.04 ± 2.1	56.81 ± 2.84
U70	40.36 ± 2.02	49.76 ± 2.49
UH50	49.13 ± 2.46	63.99 ± 3.17
UH70	51.94 ± 2.6	64.22 ± 3.21

**Table 2:** The linearities of calibration curves of flavanones.

Component	Calibration Equation	Coefficient of Determination ‘R^2^’	Coefficient of Correlation R	LOD * µg/mL	LOQ ** µg/mL
Naringin	Y = 25.500x + 6720	0.999923	0.99996	0.146	0.583
Naringenin	±Y = 33.300x + 3570	0.999924	0.99996	0.118	0.430

LOD *—limit of detection; LOQ **—limit of quantification.

## Data Availability

The data presented in this study are available on request from the corresponding author.
